# Administration of FK506 from Late Stage of Disease Prolongs Survival of Human Prion-Inoculated Mice

**DOI:** 10.1007/s13311-020-00870-1

**Published:** 2020-06-01

**Authors:** Takehiro Nakagaki, Daisuke Ishibashi, Tsuyoshi Mori, Yukiko Miyazaki, Hanae Takatsuki, Hiroya Tange, Yuzuru Taguchi, Katsuya Satoh, Ryuichiro Atarashi, Noriyuki Nishida

**Affiliations:** 1 Department of Molecular Microbiology and Immunology, Nagasaki University Graduate School of Biomedical Sciences, 1-12-4 Sakamoto, Nagasaki, 852-8523 Japan; 2 Department of Immunological and Molecular Pharmacology, Faculty of Pharmaceutical Science, Fukuoka University, 8-19-1 Nanakuma Jonan-ku, Fukuoka, 814-0180 Japan; 3 Division of Microbiology, Department of Infectious Diseases, Faculty of Medicine, University of Miyazaki, 5200 Kiyotake-cho, Miyazaki, 889-1692 Japan; 4 Department of Locomotive Rehabilitation Science, Nagasaki University Graduate School of Biomedical Sciences, 1-7-1 Sakamoto, Nagasaki, 852-8501 Japan

**Keywords:** Sporadic Creutzfeldt-Jakob disease, FK506, Microglia, Astrocyte, Spongiform change

## Abstract

Human prion diseases are etiologically categorized into three forms: sporadic, genetic, and infectious. Sporadic Creutzfeldt-Jakob disease (sCJD) is the most common type of human prion disease that manifests as subacute progressive dementia. No effective therapy for sCJD is currently available. Potential therapeutic compounds are frequently tested in rodents infected with mouse-adapted prions that differ from human prions. However, therapeutic effect varies depending on the prion strain, which is one of the reasons why candidate compounds have shown little effect in sCJD patients. We previously reported that intraperitoneal administration of FK506 was able to prolong the survival of mice infected with a mouse-adapted prion by suppressing the accumulation of abnormal prion protein (PrP) and inhibiting the activation of microglia. In this study, we tested oral administration of FK506 in knock-in mice expressing chimeric human prion protein (KiChM) that were infected with sCJD to determine if this compound is also effective against a clinically relevant human prion, i.e., one that has not been adapted to mice. Treatment with FK506, started either just before or just after disease onset, suppressed typical sCJD pathology (gliosis) and slightly but significantly prolonged the survival of sCJD-inoculated mice. It would be worthwhile to conduct a clinical trial using FK506, which has been safety-approved and is widely used as a mild immunosuppressant.

**Electronic supplementary material:**

The online version of this article (10.1007/s13311-020-00870-1) contains supplementary material, which is available to authorized users.

## Introduction

Sporadic Creutzfeldt-Jakob disease (sCJD) accounts for about 75% of human prion diseases and leads to death through rapidly progressing dementia and akinetic mutism [1, 2]. Histologically, human prion diseases are characterized by massive brain atrophy accompanied with spongiosis, gliosis, and accumulation of abnormally aggregated prion protein (PrP^Sc^) [3, 4]. It has been proposed that the conformational conversion of normal prion protein (PrP^C^) to abnormal form (PrP^Sc^) in neurons plays a central role in sCJD pathogenesis. Some drugs, such as pentosan polysulfate (PPS) [5] and quinacrine [6], inhibit this conversion process of PrP and they have been proposed as potential therapeutic agents for prion diseases because of their effects in prion-infected mouse models. However, the corresponding clinical trials on sCJD patients did not show any improvement in either patient symptoms or survival periods [7–9]. Doxycycline has also been reported to prolong survival periods of PrP^Sc^-inoculated mice [8]; however, its effect in humans appears to be limited to patients with early-stage sCJD [10].

There are several reasons that can explain the difficulty in developing therapeutics for sCJD. First, anti-prion drugs only show effects on animal prions in experimental models when the administration of drugs was started at the same time as prion infection, indicating that the drugs may inhibit the propagation of prions [11, 12]. However, the human trials of these drugs were conducted only after the disease was established because early definitive diagnosis has yet to be developed [13]. Second, drug effects will be dependent on the prion strain. Prions exhibit strain-diversity and the mechanisms for this diversity are still unknown. Notably, the Rocky Mountain Laboratory (RML) strain, a mouse-adapted scrapie prion, is often used in testing therapeutics, but its biochemical and pathological properties are distinct from those of human sCJD prions [14, 15]. Therefore, even a compound that doubles the survival period of prion-inoculated mice may not necessarily be effective for treating sCJD. This problem has been previously described [16]. To evaluate therapeutic effects against human prions, drug-evaluation can be conducted using sCJD prion-inoculated mice. Another possible reason for the failure of previous clinical trials may be more fundamental. Most anti-prion compounds inhibit PrP-conversion in prion-infected cells. However, the direct neurotoxicity of PrP^Sc^ is not clear and reduction of PrP^Sc^ may not be enough to stop neuronal loss if the pathological reactions have already started. For example, microglia have important roles in protecting neurons but over-activated microglia can induce neuronal cell death by releasing pro-inflammatory cytokines [17–20].

We have previously reported that the immunosuppressant, FK506, can prolong survival periods of mice infected with a mouse-adapted prion strain, Fukuoka-1, by regulating both glial activation and the activation of neuronal autophagy. This resulted in reduced PrP^Sc^ accumulation [21]. To elucidate the effect of FK506 on human prions, we administered the compound to sCJD prion-inoculated mice.

## Materials and Methods

### Reagents

3F4 antibody (BioLegend, San Diego, USA) is a mouse monoclonal antibody recognizing the amino acid residues 109–112 of human PrP. Anti-ionized calcium-binding adapter molecule 1 (IBA1) antibody to detect microglia (Wako Pure Chemical Industries, Japan) and anti-glial fibrillary acidic protein (GFAP) antibody to detect astrocytes (DAKO, Japan) are rabbit polyclonal antibodies. FK506 (Selleck Chemicals, Houston, USA) was dissolved as previously described [22]. Briefly, FK506 was mixed with Kolliphor HS15 (gifted from BASF Japan), tetraglycol (Sigma-Aldrich, Japan), and ethyl oleate (Nacalai Tesque, Japan) and was stored at 25 °C. Doxycycline hyclate (Sigma-Aldrich) was dissolved in dH_2_O and stored at − 20 °C.

### Brains of sCJD Patients

**Fig. 1:**
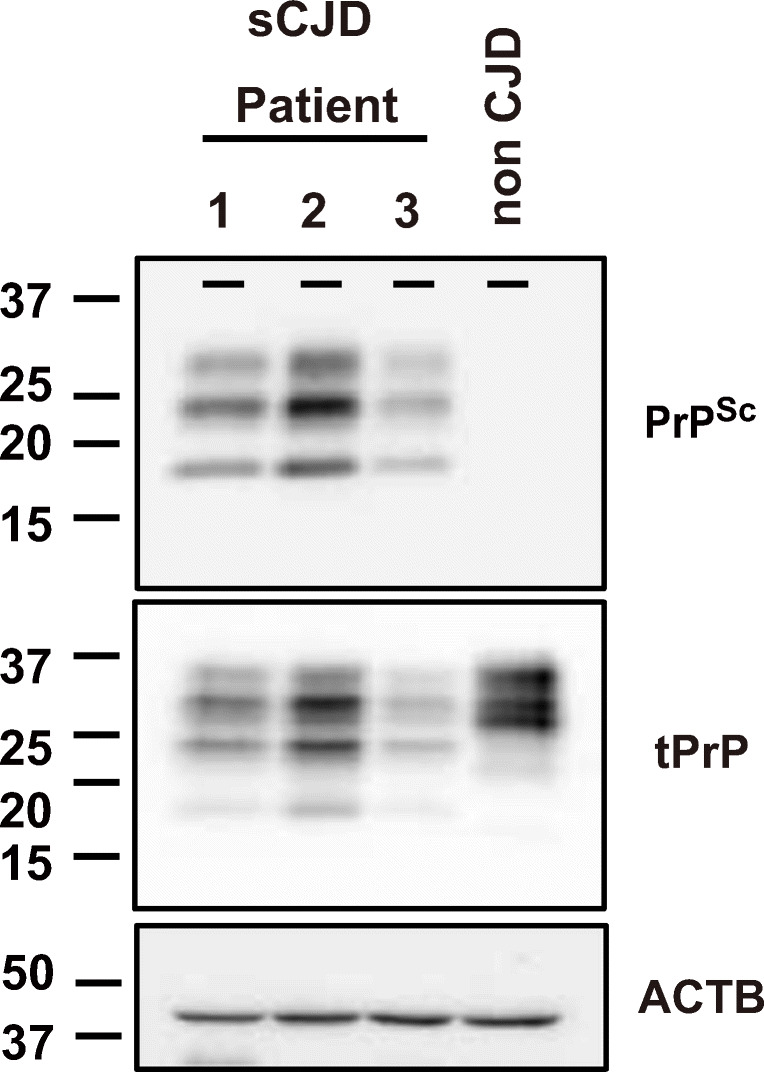
Detection of PrP^Sc^ and tPrP from brain homogenates of sCJD patients. PrP^Sc^ and total PrP (tPrP) in three sCJD patients were detected by Western blotting with the 3F4 antibody. PrP^Sc^ and total tPrP are shown in the upper and middle panels, respectively. β-Actin was used as an internal control (lower panel)

All three patients were female and diagnosed as having classical-type sCJD (sCJD MM1) according to Parchi’s classification [23]. Patient 1 died at 75 years old [24]. Patients 2 and 3 died at 67 and 71 years old, respectively (Fig. 1 and Table 1) [25]. Tissue samples of brains were homogenized in 10% (w/v) phosphate-buffered saline (PBS; Nacalai Tesque, Japan) using a multi-bead shocker (Yasui Kikai, Japan) [25], and supplemented with a protease inhibitor mixture (Roche, Japan). Informed consents were obtained from patients and/or patient families.

**Table 1:** Information of sCJD patients used for inoculum

Patient No.	CJD type	Sex	Age at death (years old)	Intensity of PrP^Sc^	Reference
1	MM1	Female	71	1	(25)
2	MM1	Female	67	1.71	(25)
3	MM1	Female	75	0.54	(24)

### Animal Models

Five-week-old knock-in mice expressing human and mouse chimeric PrP (KiChM), which were described elsewhere [25, 26], were inoculated intracerebrally with 20 μl of brain homogenate (BH) from a sCJD patient. Mice were monitored daily until the terminal stage of the disease or sacrificed at an indicated time point. Clinical onset was defined as the weight of mice falling lower than 28 g, which is about 5 g less than that of uninoculated mice, or as the appearance of any sCJD symptom, such as priapism, hunchback, ataxic gait, and abnormal reflex with non-parallel hind limbs. Clinical scores were first graded by body weight (BW) of mice: 0, healthy; 1, 26–28 g; 2, 24–26 g; 3, 22–24 g; 4, 20–22 g; 5, less than 20 g; 6, death. The score was then added one when the mice showed mentioned symptoms (Additional file 1). Mice weighed less than 22 g with at least two symptoms were euthanized under anesthesia and their clinical score was recorded as 6 (death). Some of the mice were sacrificed at 140 d.p.i. After brains were removed, the right hemispheres were frozen and homogenized at 20% (w/v) in PBS to conduct Western blotting. Total proteins were extracted by mixing the samples with an equal volume of lysis buffer (0.5% Triton X-100, 0.5% deoxycholic acid, 150 mM NaCl, 25 mM Tris-HCl, pH 7.5). The left hemispheres were fixed in 10% neutral-buffered formalin (WAKO) to analyze histopathological changes.

### Administration of FK506

In mice inoculated with sCJD prion from either patient 1 or patient 2, FK506 was orally administered at 1.0 mg/kg/day from 110 or 140 d.p.i. In the case of mice inoculated with BH from sCJD patient 3, FK506 was started from 135 d.p.i. with the lower concentration of the drug which adjusted as 0.1 mg/kg/day.

### Western Blotting

Total protein concentrations were measured using a Protein Assay Bicinchoninate Kit (Nacalai). To detect PrP^Sc^, the samples were digested with 20 μg/ml of protease K (PK) for 30 min at 37 °C. Loading buffer (50 mM Tris-HCl [pH 6.8], containing 5% (v/v) glycerol, 1.6% (w/v) sodium dodecyl sulfate [SDS], and 100 mM dithiothreitol) was added to the proteins, and the mixtures were incubated at 95 °C for 10 min. SDS polyacrylamide gel electrophoresis was performed using 15% (w/v) acrylamide gels. The proteins were transferred onto an Immobilon-P membrane (Merck, Japan) in transfer buffer containing 20% (v/v) methanol, and the membrane was blocked with 5% (w/v) nonfat dry milk in tris-buffered saline with Tween 20 (TBST, 10 mM Tris-HCl [pH 7.8], 100 mM NaCl, 0.1% [v/v] Tween 20) for 1 h before blotting with the primary antibody overnight at 4 °C. Immunoreactive bands were visualized using Clarity Western ECL substrate (Bio-Rad, Japan).

### Quaking-Induced Conversion Assay

Ninety microliter of reaction buffer and 10 μ L of serially diluted BH was loaded into 96-well black plate with clear bottom (Greiner, Japan). The composition and final concentration of each in reaction buffer was 500 mM NaCl, 50 mM PIPES (pH 7.0), 1 mM EDTA, 0.001% SDS 0.01 mM thioflavin T (ThT), and 0.1 mg/mL recombinant human PrP (residues 23-231). Ten percent BHs used for seeds were serially diluted 10^−2^ to 10^−9^. The plate was sealed (plate sealer, Nalgene Nunc International) and incubated at 42 °C in a Thermomixer C (Eppendorf, Japan) with cycles of 1-min shaking (1000 rpm double orbital) and 1-min incubation. ThT fluorescence was measured with infinite F200 PRO (Tecan, Japan) (430 ± 20-nm excitation and 485 ± 20-nm emission; bottom read) at 48 h after the reaction was started. The fluorescent value larger than average values of uninoculated mice brains plus three standard deviations was determined positive. The 50% positive reactivity (50% of seeding dose: SD_50_) was calculated using the Spearman–Kärber method [27].

### Histochemistry

The fixed hemispheres were embedded in paraffin and sectioned into 3-μm slices. Tissue sections were stained with hematoxylin and eosin. For IBA1 and GFAP staining, after deparaffinization and rehydration, the sections were treated with 0.3% (v/v) hydrogen peroxidase in methanol for 30 min to inactivate endogenous peroxidase and then incubated with 3% nonfat dry milk in TBST for 60 min at room temperature. The blocked sections were subsequently reacted with primary antibody overnight at room temperature, then reacted with the Envision polymer horseradish peroxidase (HRP)–conjugated anti-rabbit immunoglobulin G antibodies (DAKO) for 60 min at room temperature. Immunostaining was visualized using 3,3′-diaminobenzidine (DAB; Dojindo Lab, Japan). The hydrolytic autoclaving and formic acid method for PrP^Sc^ staining was performed as described previously [21].

### Statistical Analysis

An unpaired *t* test was used for comparison between two groups. The log-rank test was used for analyzing the survival time. All statistical analyses were performed using GraphPad Prism software.

### Ethical Approval

All animal experiments were approved by the Ethics Committee of Nagasaki University and were performed under the Guidelines for Animal Experimentation of Nagasaki University. These experiments also confirmed to the recommendations issued in the Guide for the Care and Use of Laboratory Animals by the National Institutes of Health.

## Results

### Comparison of the Therapeutic Effects of FK506 and Doxycycline in a sCJD-Inoculated Mouse Model

**Fig. 2:**
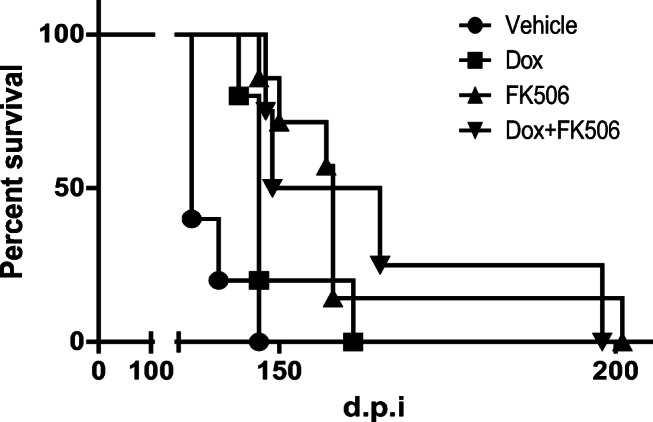
Survival curves of sCJD-1-inoculated mice treated with FK506 and doxycycline. The survival curves of sCJD-1-inoculated mice that were administered with 0.1 mg/kg/day of FK506 (FK) and/or 2 mg/kg/day of doxycycline (Dox) from disease onset (130 d.p.i.). The survival of the groups treated with vehicle, Dox alone, FK506 alone, and combined therapy (Dox and FK506 together) are indicated by circles, square, upright triangles, and upside-down triangles, respectively

When wild-type mice were inoculated with BH from a sCJD patient, they did not show any symptoms. In contrast, KiChM mice were susceptible to human prions and died about 150 days post-inoculation (d.p.i.) when they are intracerebrally inoculated with sCJD prion [25, 26]. Doxycycline (Dox), which promotes the degradation of PrP^Sc^, has been one of the most promising candidate therapeutic agents for sCJD [8]. To investigate and compare the therapeutic effects of FK506 and Dox, KiChM mice were inoculated with 10% BH from patient no. 1 (sCJD-1 in Fig. 1 and Table 1). When symptoms appeared in the mice from 130 d.p.i., they were administered 0.1 mg/kg/day FK506 or 2.0 mg/kg/day Dox or vehicle. The mice-administered Dox lived until 149.2 ± 6.7 d.p.i., which was about 10 days longer than the vehicle-treated group (*p* < 0.05). The mice-administered FK506 survived until 161.8 ± 18.0 d.p.i., which was about 20 days longer than the vehicle-treated group (Fig. 2 and Table 2). The mice administered with both FK506 and Dox lived until 165 ± 23.3 d.p.i. They lived significantly longer than vehicle and Dox-only groups, but for almost the same survival period as the FK506-only group.

**Table 2:** Comparison of therapeutic effect of FK506, doxycycline, and combined therapy on sCJD-inoculated mice

Inoculum (patient no.)	Group	Start point (d.p.i)	Number	Mean ± SD (days)	*p* value (v.s. vehicle)
1	Vehicle		5	139.8 ± 4.4	
	Doxycycline	130	5	149.2 ± 6.7	< 0.05
	FK506	130	7	161.8 ± 18.0	< 0.01
	Dox + FK506	130	4	165.0 ± 23.3	< 0.01

The survival periods of these mice are shown. Statistical significance was determined by a log-rank test. ± indicates SD.

### FK506 Suppresses the Progression of Symptoms and Prolongs the Survival Period of sCJD Prion-Inoculated Mice

**Fig. 3:**
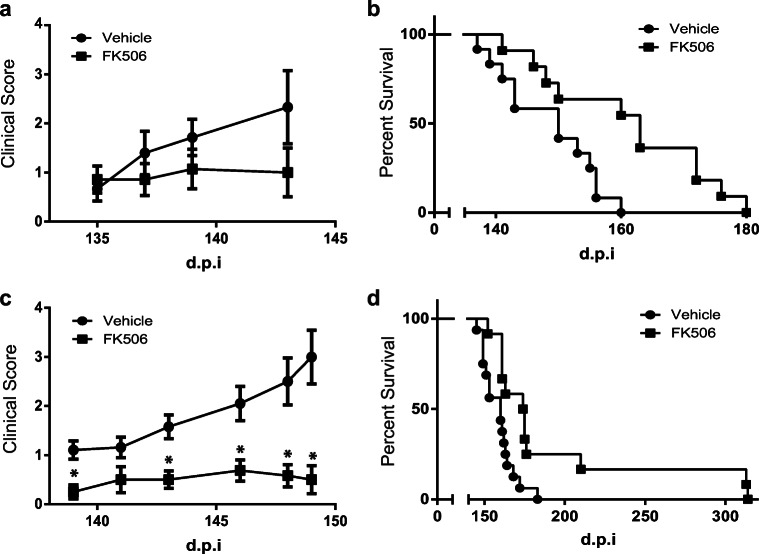
Clinical scores and survival curves of sCJD prion-inoculated mice. Clinical scores were recorded from 140 d.p.i until the first mouse died. Survival periods were also compared between the vehicle-treated control mice (circles) and FK506-treated mice (squares) from 110 d.p.i. **a** Clinical scores of FK506- or vehicle-treated mice 110 days after they were inoculated with sCJD-2 prion. **b** Survival curves of these mice. **c** Clinical scores of FK506- or vehicle-treated mice 110 days after they were inoculated with sCJD-3 prion. **d** Survival curves of these mice. Statistical significance of differences in clinical scores was determined using a two-tailed Student *t* test, and that of differences in the survival curves was determined using log-rank test. *: *p* < 0.001 compared with control group. Error bars indicate standard error of the mean (SEM)

To investigate the effect of FK506 on sCJD prion-inoculated model, KiChM mice with sCJD prion were administered vehicle or FK506 (1.0 mg/kg/day) either from 110 d.p.i. (just before disease onset) or from 140 d.p.i. (after the symptoms appeared), and symptom scores were recorded (see Additional File 1). When we inoculated KiChM mice with BH from patient no. 2 (sCJD-2 in Fig. 1 and Table 1), the deterioration in symptom scores from 135 to 145 d.p.i. in mice that received FK506 from 110 d.p.i were suppressed (Fig. 3a).

After treatment with FK506 beginning at 110 d.p.i., symptom scores of mice inoculated with BH from patient no. 3 (sCJD-3 in Fig. 1) were better than those of vehicle-treated mice (Fig. 3c). In addition, both groups inoculated with sCJD-2 and sCJD-3 prion survived 12 and 30 days longer than the untreated control, respectively (Fig. 3b, d and Table 3). Moreover, mice inoculated with sCJD-3 prion survived significantly longer than vehicle-treated group even when the treatment was started from 140 d.p.i (Table 3).

**Table 3:** Therapeutic effects of FK506 on KiChM mice inoculated with sCJD prion

Inoculum (patient no.)	FK506 (mg/kg/day)	Start point (d.p.i)	Number	Mean ± SD (days)	*p* value
2	0		12	148.6 ± 7.7	
	1.0	110	11	161.0 ± 13.2	< 0.05
	1.0	140	9	154.8 ± 5.0	n.s
3	0		16	158.9 ± 10.0	
	1.0	110	12	194.6 ± 57.4	< 0.01
	1.0	140	8	172.3 ± 11.5	< 0.05

The survival periods of these mice are shown. Statistical significance was determined by a log-rank test. ± indicates SD. n.s indicates no significance.

### FK506 Suppresses the Activation of Glial Cells and Spongiform Change

**Fig. 4:**
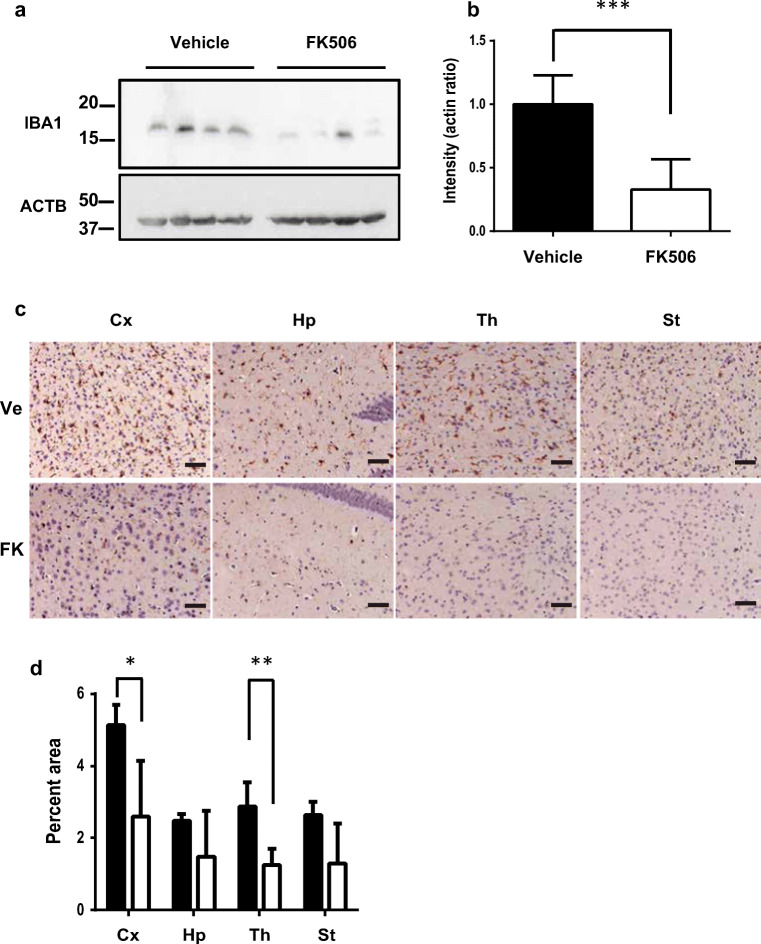
Comparison of microglia in sCJD-3 prion-inoculated mice. Some of the sCJD-3-inoculated mice that had been treated with FK506 or vehicle from 110 d.p.i were sacrificed at 140 d.p.i. **a** Western blot analysis of the expression level of IBA1. β-Actin (ACTB) was used as an internal control. **b** Band intensities of samples from FK506-treated mice are expressed as a percentage of those of the control mice. The results in the graph are the mean ± SD. **c** IBA-1-positive cells in the cortex (Cx), hippocampus (Hp), and thalamus (Th) were visualized by immunohistochemical staining. **d** The percentages of occupied by IBA-1-positive cells were calculated and compared between the FK506-treated group (FK) and the vehicle-treated group (Ve). Scale bars represent 100 μm. Statistical significance was determined using a two-tailed Student *t* test. *: *p* < 0.05, **: *p* < 0.01 compared with the control. Error bars indicate standard deviation (SD)

After the 30-day administration of FK506 or vehicle (140 d.p.i), we collected brains from sCJD-3 prion-inoculated mice and evaluated the degree of glial activation. IBA1 [17], which is also called allograft inflammatory factor-1 (AIF-1), was assessed as a marker of activated microglia [18]. The expression levels of IBA1 in whole brains of mice at 140 d.p.i were analyzed by Western blotting. The levels of IBA1 in whole brains of FK506-treated mice were significantly lower than those of vehicle-treated mice (Fig. 4a, b). Immunohistochemistry revealed that the areas occupied by microglia in the cortex and thalamus were significantly smaller in FK506-treated mice compared with those in vehicle-treated mice, but there was no significant difference in the hippocampus or striatum (Fig. 4c, d).

**Fig. 5:**
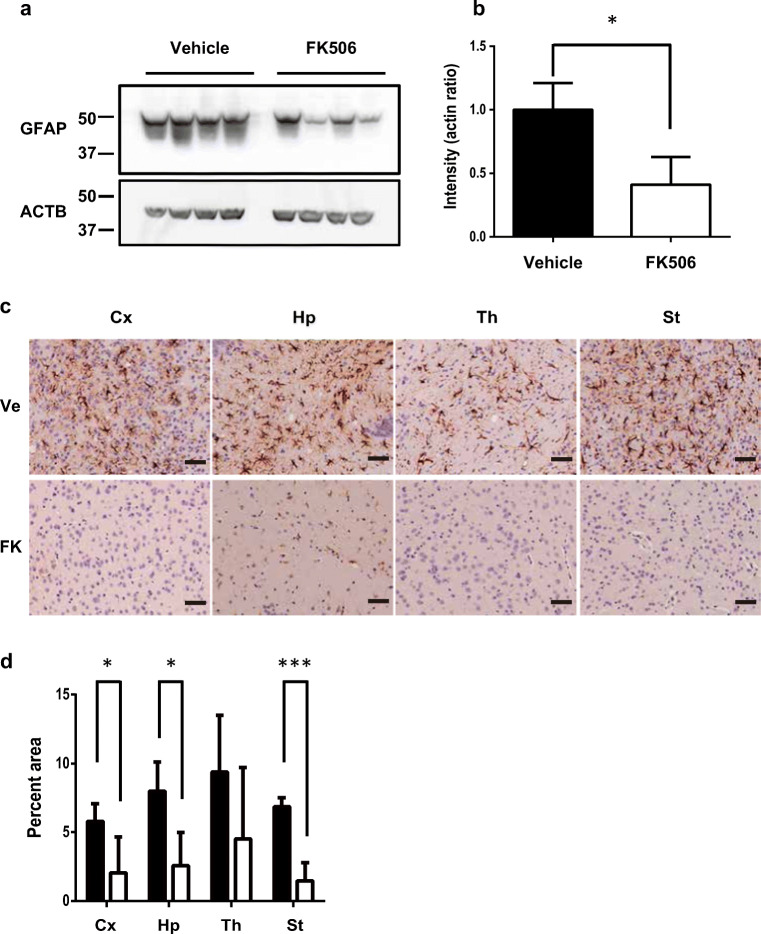
Comparison of astrocyte in sCJD-3 prion-inoculated mice. **a** Western blot analysis of the expression level of GFAP. ACTB was used as an internal control. **b** Band intensities of samples from FK506-treated mice are expressed as a percentage of those of the control mice. The results in the graph are the mean ± SD. **c** GFAP-positive cells were visualized by immunohistochemical staining. **d** The percentages of occupied by IBA-1-positive cells were calculated and compared between the FK506-treated group (FK) and the vehicle-treated group (Ve). Scale bars represent 100 μm. Statistical significance was determined using a two-tailed Student *t* test. *: *p* < 0.05, **: *p* < 0.01 compared with the control. Error bars indicate standard deviation (SD)

Next, we analyzed the expression levels of GFAP at 140 d.p.i. [28]. The levels of GFAP in the brains of FK506-treated mice were significantly lower than those of vehicle-treated mice (Fig. 5a, b). The areas occupied by astrocytes in the cortex, hippocampus, and striatum of the FK506-treated group were less than half those of the vehicle-treated group (Fig. 5c, d).

**Fig. 6:**
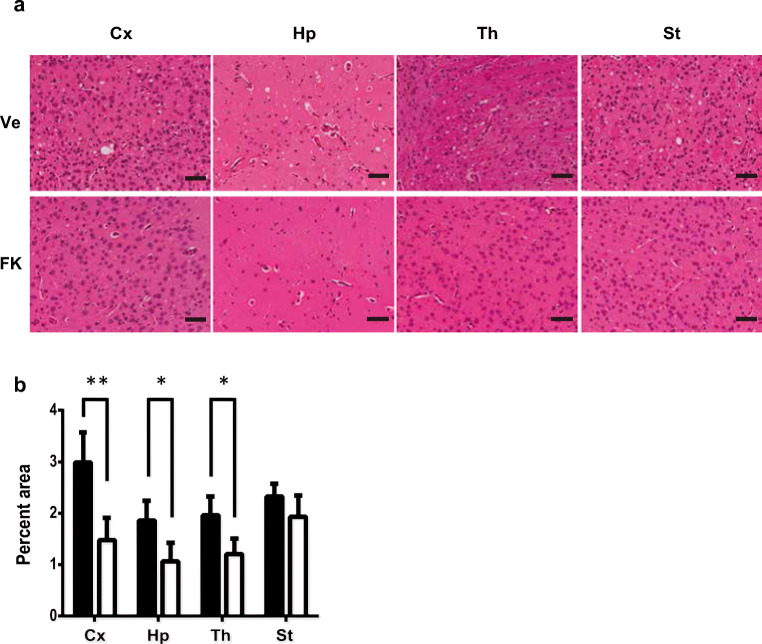
Comparison of the degree of vacuolation in sCJD-3 prion-inoculated mice. **a** The vacuole areas in the brains of mice treated with FK506 or vehicle 110 d.p.i in sCJD-3-inoculated groups were visualized by staining with hematoxylin and eosin (HE staining) at 140 d.p.i. Ve and FK indicate the mice they were treated with vehicle or FK506 respectively. **b** The percentages of the vacuolated areas were calculated and compared between the FK506-treated group and the vehicle-treated group. Statistical significance was determined using a two-tailed Student *t* test. *: *p* < 0.05, **: *p* < 0.01, ***: *p* < 0.001 compared with the control. Error bars indicate SD

The spongiform areas in the cortex, hippocampus, thalamus, and striatum were analyzed by staining brain sections with hematoxylin and eosin and calculating the percentage of the vacuolated area in each brain region. In FK506-treated mice inoculated with sCJD-3 prion, the percentages of vacuolated areas were lower than those in control mice, particularly in the cortex, hippocampus, and thalamus (Fig. 6).

### Comparison of the Accumulation of PrP^Sc^ and Total PrP at 140 d.p.i and Each Terminal Stage

**Fig. 7:**
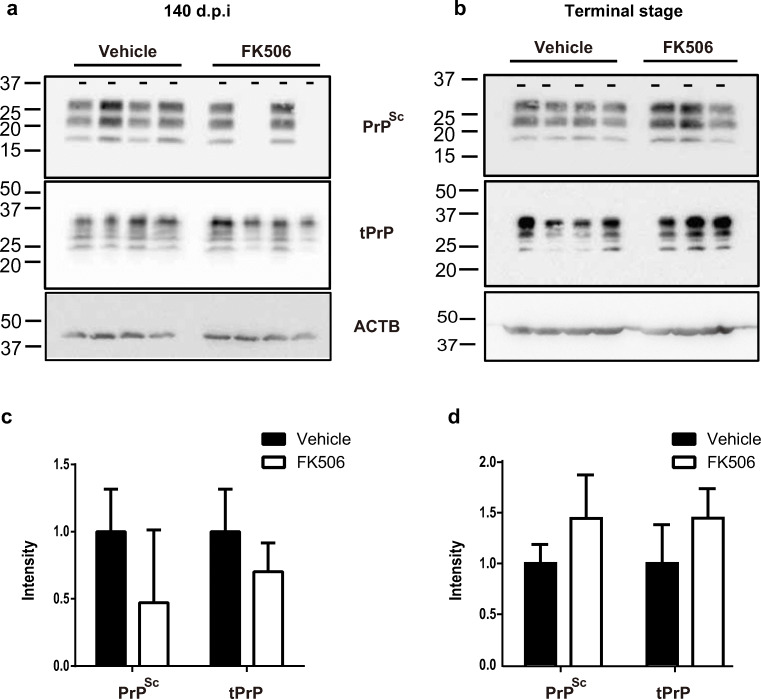
Comparison of the amount of PrPSc in the brains of sCJD-3 prion-inoculated mice. **a**, **b** PrP^Sc^ and tPrP in the brains of mice treated with FK506 or vehicle from 110 d.p.i in sCJD-3-inoculated groups were detected at 140 d.p.i (**a**) and each terminal stage (**b**). **c**, **d** Band intensities of samples from FK506-treated mice are expressed as a percentage of those of the control mice. The results in the graph are the mean ± SD. β-Actin (ACTB) was used as an internal control for evaluating the amount of tPrP

In mice inoculated with sCJD-3 prion, PrP^Sc^ was not detected in two of the four FK506-treated mice, whereas it was detected in the brains of all mice in the vehicle-treated group by Western blotting at 140 d.p.i. (Fig. 7a, c). In the two PrP^Sc^-negative cases, we also evaluated the amount of seeding activity in the brains by using the endpoint quaking-induced conversion (QUIC) method. Their SD_50_ was about 1/1000 of the control group (Additional file 2). At each terminal stage, the FK506-treated mice tended to accumulate more PrP^Sc^ than the control group, although there was no significant difference (Fig. 7b, d).

## Discussion

Orally administered FK506 prolonged the survival of sCJD prion-inoculated mice expressing humanized PrP. The treatment suppressed glial cell activation and spongiform changes. These results indicate that FK506 administration possibly delays the progression of pathological damage, resulting in prolonged survival. To our knowledge, this is the first report of successfully treating sCJD in a humanized mouse model.

FK506 has already been used in humans as an immunosuppressant to treat autoimmune diseases and to prevent rejection of organ transplantation and graft versus host disease (GVHD) [29–32]; furthermore, its side effects and pharmacokinetics are predictable. Administering FK506 at 1.0 mg/kg/day to a mouse is equivalent to 0.081 mg/kg/day for a human, based on a “human equivalent dose” (HED) estimation [33]. FK506 for kidney transplantation patients typically starts at 0.3 mg/kg/day and gradually reduces to 0.12 mg/kg/day, a dose that is taken for years. Therefore, the doses of FK506 used in this study are acceptable for long-term administration to sCJD patients.

Doxycycline treatment also prolonged the survival of mice inoculated with sCJD-1 prion but its effect on survival was less than that of FK506. The survival period of mice receiving FK506 and Dox combination therapy was almost the same as that of the mice receiving FK506 only. Although further experiments are needed, these results indicate that FK506 has potential to improve the symptoms and survival of sCJD patients even after onset of the disease.

In the case of sCJD-3 prion-inoculated mice, accumulation of PrP^Sc^ was suppressed in two of four treated mice, whereas it was not inhibited in the other mice at 140 d.p.i. This result is very surprising but should be carefully considered. We have previously reported that FK506 inhibits prion disease progression by promoting PrP^Sc^ degradation and inhibiting the proliferation and/or activation of microglia when treated from an early stage of prion infection [21]. On the other hand, other groups reported that administration of FK506 from after symptomatic stage did not affect the amount of PrP^Sc^ [34, 35]. These results suggest that FK506 can suppress the accumulation of PrP^Sc^ when its amount is relatively low but cannot suppress the accumulation of large amount of PrP^Sc^. In our experiments, the rate of PrP^Sc^ accumulation might be more likely to differ between individuals when KiChM mice were inoculated with human BHs at relatively low concentrations comparing to wild-type mice inoculated with mouse adapt-prions (Table 2). In particular, the period just before disease onset, such as 110 d.p.i. in this experiment, might be a time when PrP^Sc^ have begun to accumulate explosively, and individual differences in the accumulation of PrP^Sc^ are likely to occur. For these reasons, administration of FK506 could suppress the accumulation of PrP^Sc^ only in the mice with relatively low amount of PrP^Sc^ at 110 d.p.i. It is preferable to start treatment after estimating the amount of PrP^Sc^ using the QUIC method in further examination. It will be helpful to solve this problem if we can collect enough volume of central spinal fluid from living mice for QUIC.

The amount of PrP^Sc^ in the brain of sCJD patient 2 was about twice as that of sCJD patient 3 (Fig. 1). Then, clinical score of vehicle group of mice inoculated with sCJD-2 prion was significantly worse than that of mice inoculated with sCJD-3 prion (2.33 ± 0.91 vs 1.1 ± 1.91 *p* value < 0.05). For this reason, the administration of FK506, beginning after obvious symptoms were observed, prolonged the survival of sCJD-3 prion-inoculated, but not of sCJD-2 prion-inoculated mice. Therefore, we predict that administration of FK506 from the early stage of the disease suppresses not only gliosis and vacuolation but also the accumulation of PrP^Sc^. In other words, the early diagnosis of sCJD using QUIC method [36], molecular probes of abnormal proteins [37, 38], and MRI [39] is critical.

In recent decades, the main strategy for developing drugs against prion diseases focused on reducing abnormal PrP accumulation using either small compounds, antibodies against PrP, or siRNA to stop PrP expression. These treatments showed certain effects on prion-inoculated mice [40–42]. These findings indicate that the conversion of PrP has an important role in the initial pathogenesis, and that anti-PrP compounds prolong the incubation time of the disease. The preventive use of anti-PrP treatments will be especially beneficial for genetic human prion diseases. However, inhibiting the conversion may be insufficient for patients who have already shown symptoms, because the therapeutic effects of pentosan polysulfate and quinacrine for the patients have been restricted [5, 6].

Glial cells protect neurons by removing aggregated proteins and releasing anti-inflammatory cytokines [20, 43], but they can also exacerbate the disease state by inducing neuroinflammation in several neurodegenerative models including prion diseases [44, 45]. In the sCJD MM1-inoculated mice model, the expression levels of activated glial markers have been upregulated from early clinical stage [46]. Therefore, when treatment is initiated after onset, we predict that modulation of microglia and astrocytes is very important for prolonging the survival of mice with sCJD prion.

Several biological mechanisms have recently been reported as potential therapeutic targets for prion diseases. Stimulation of innate immunity [47–49] and autophagy [21, 50, 51] can reduce the amount of PrP^Sc^ in infected neurons or brains. The unfolded protein response (UPR) is also over-activated in prion diseases and promotes disease progression, and UPR inhibitors can restore memory loss and extend the survival of prion-inoculated mice [52, 53]. Recently, sCJD prion-infected cells were developed [54, 55]. Using these cells and humanized mice will enable the effects of these new candidates against human prions to be assessed. Although further work is needed to elucidate the details of the mechanism of FK506 action, data indicate that the effect will not depend on prion strain and oral administration of FK506 has good potential as a therapeutic for sCJD. It is worth considering the establishment of an appropriate combination therapy with other drugs that act through various anti-prion mechanisms.

## Electronic Supplementary Material

ESM 1 Additional file 1: Criteria of clinical scores. Criteria for assigning clinical scores are shown. These scores were determined by animal body weight and the existence of symptoms, such as priapism, hunchback, ataxic gait, and non-parallel hind limbs. Additional file 2: Comparison of Intensity of PrP^Sc^ and SD_50_ in the each brains of mice inoculated with sCJD-3 prion. The intensity and SD_50_ at 140d.p.i in the brains of mice inoculated with sCJD-3 prion were measured by Western blotting and QUIC, respectively. (PDF 171 kb).

## Data Availability

All data generated or analyzed during this study are included in this published article.
