# Supplementary material for: Administration of FK506 from Late Stage of Disease Prolongs Survival of Human Prion-Inoculated Mice
Source: Neurotherapeutics. 2020 Jun 1;17(4):1850–60. doi: 10.1007/s13311-020-00870-1 (PMC7851258; doi:10.1007/s13311-020-00870-1)
Supplement: Supplementary file 1 — Additional file 1: Criteria of clinical scores. Criteria for assigning clinical scores are shown. These scores were determined by animal body weight and the existence of symptoms, such as priapism, hunchback, ataxic gait, and non-parallel hind limbs. Additional file 2: Comparison of Intensity of PrPSc and SD50 in the each brains of mice inoculated with sCJD-3 prion. The intensity and SD50 at 140d.p.i in the brains of mice inoculated with sCJD-3 prion were measured by Western blotting and QUIC, respectively. (PDF 171 kb). [file 13311_2020_870_MOESM1_ESM.pdf]

Additional file. 1 Criteria of clinical scores

Score	Definition
0	28g ≤BW without any symptoms
1	26g ≤ BW <28g, 28g ≤ BW with one or more symptom
2	24g ≤ BW < 26g, 26g ≤ BW with one or more symptom
3	22g ≤ BW < 24g, 24g ≤ BW with one or more symptom
4	20g ≤ BW < 22g
5	Less than 20g
6	Death

Additional file. 2 Comparison of Intensity of PrP^{Sc} and SD50 in the each brains of mice inoculated with sCJD-3 prion

	Intensity	Log SD50 (g tissue)
Vehicle	0.803	9.25
	1.348	8.75
	0.733	9.25
	1.117	8.88
FK506	0.963	9.25
	0.018	6.63
	1.065	9.50
	0.005	6.25
